# Elevated Concentrations of Serum Immunoglobulin Free Light Chains in Systemic Lupus Erythematosus Patients in Relation to Disease Activity, Inflammatory Status, B Cell Activity and Epstein-Barr Virus Antibodies

**DOI:** 10.1371/journal.pone.0138753

**Published:** 2015-09-24

**Authors:** Anette H. Draborg, Magnus C. Lydolph, Marie Westergaard, Severin Olesen Larsen, Christoffer T. Nielsen, Karen Duus, Søren Jacobsen, Gunnar Houen

**Affiliations:** 1 Department of Autoimmunology and Biomarkers, Statens Serum Institut, Copenhagen, Denmark; 2 Department of Infectious Diseases and Rheumatology, Rigshospitalet, Copenhagen University Hospital, Copenhagen, Denmark; University of Texas Health Science Center at Houston, UNITED STATES

## Abstract

**Objective:**

In this study, we examined the concentration of serum immunoglobulin free light chains (FLCs) in systemic lupus erythematosus (SLE) patients and investigated its association with various disease parameters in order to evaluate the role of FLCs as a potential biomarker in SLE. Furthermore, FLCs’ association with Epstein-Barr virus (EBV) antibodies was examined.

**Methods:**

Using a nephelometric assay, κFLC and λFLC concentrations were quantified in sera from 45 SLE patients and 40 healthy controls. SLE patients with renal insufficiency were excluded in order to preclude high concentrations of serum FLCs due to decreased clearance.

**Results:**

Serum FLC concentrations were significantly elevated in SLE patients compared to healthy controls (p<0.0001) also after adjusting for Ig levels (p<0.0001). The concentration of serum FLCs correlated with a global disease activity (SLE disease activity index (SLEDAI)) score of the SLE patients (r = 0.399, p = 0.007). Furthermore, concentrations of FLCs correlated with titers of dsDNA antibodies (r = 0.383, p = 0.009), and FLC levels and SLEDAI scores correlated in the anti-dsDNA-positive SLE patients, but not in anti-dsDNA-negative SLE patients. Total immunoglobulin (IgG and IgA) concentrations correlated with FLC concentrations and elevated FLC levels were additionally shown to associate with the inflammatory marker C-reactive protein and also with complement consumption determined by low C4 in SLE patients. Collectively, results indicated that elevated serum FLCs reflects increased B cell activity in relation to inflammation. SLE patients had an increased seropositivity of EBV-directed antibodies that did not associate with elevated FLC concentrations. An explanation for this could be that serum FLC concentrations reflect the current EBV activity (reactivation) whereas EBV-directed antibodies reflect the extent of previous infection/reactivations.

**Conclusion:**

SLE patients have elevated concentrations of serum FLCs that correlate with global disease activity scores and especially serologic markers for active disease. These findings are suggestive of circulating FLCs having potential as a new supplementary serologic biomarker in SLE.

## Introduction

Systemic lupus erythematosus (SLE) is an important autoimmune disease with a prevalence of 20–150 cases per 100.000 [1,2], and typically presents in women (90% of cases) in the reproductive age. The typical course of the disease is illustrated by periods of disease flares alternating with remission. The etiology of SLE is believed to be multi-factorial with genetic and environmental factors, both contributing to the development of the disease [1,3–5].

B cell activation is central in the disease mechanism of SLE, as it is characterized by the production of autoantibodies against conserved cellular components including double-stranded DNA (dsDNA) (58–70% of SLE patients [3,6]), histones, Ro52, Ro60, La and Sm [4,6–8]. Furthermore, extensive polyclonal B cell hyperactivity is observed during active disease [9,10].

During antibody synthesis in plasma cells, up to 40% excess immunoglobulin (Ig) light chains are produced in proportion to Ig heavy chains. Free light chains (FLCs) are released to the circulation; κFLCs are released as either monomers or dimers and λFLCs are released as dimers. The serum FLCs are rapidly catabolized by the kidneys with a half life in the circulation of 2–4 hours for κFLCs and 3–6 hours for λFLCs. The normal concentration of FLCs in serum and urine is kept at low levels due to the kidneys massive capacity for clearing of serum FLCs (10–30 grams/day). Thus, in individuals with normal renal function, increased concentrations of serum FLCs are presumably a result of increased plasma cell activity [11–13]. However, some SLE patients suffer from glomerulonephritis and renal insufficiency and as a consequence these patients presumably have decreased clearance of FLCs and thereby an elevated concentration in the circulation.

Previous studies on FLC levels in SLE patients have revealed elevated levels of FLCs in both serum and urine in periods of active disease [12–17]. Three previous studies have demonstrated elevated concentrations of serum FLC using a quantitative nephelometric assay. Aggarwal *et al*. investigated FLC concentrations in serum from 74 SLE patients and showed significantly increased concentrations compared to healthy controls and also found a correlation between serum FLC levels and SLEDAI scores [14]. Jolly *et al*. quantified the concentrations of FLCs in serum from 77 SLE patients and observed a correlation between λFLC and both SLEDAI scores and PGA (physician global assessment), and actually, λFLC concentrations accounted for 31% of the variance in SLEDAI scores of SLE patients [16]. Chiche *et al*. found that 8 out of 11 SLE patients had elevated concentrations of serum FLC, but all had normal κ:λ ratios [15]. In addition, Hopper *et al*. applied a radioimmunoassay and found elevated FLC concentrations in urine of SLE patients during active disease [12,13].

Many studies have linked Epstein-Barr virus (EBV) infection to SLE [18–22]. EBV infection during childhood is asymptomatic, but infection in adolescence causes infectious mononucleosis in 30–70% of cases [23,24]. After primary infection, EBV persists in latent form within immortalized memory B cells [25–27]. Occasionally, EBV reactivates and switches to lytic cycle and thereby the infected memory B cells undergo differentiation into plasma cells, activating viral and cellular promoters resulting in viral gene expression and viral genome replication [28]. This gives rise to release of virus that can infect other B cells [27].

Nearly all SLE patients (99.5%) and a large proportion of healthy adults (94.5%) are infected with EBV [29]. Studies have shown an increased number of latently EBV-infected cells [18] and an abnormally high viral load in the peripheral blood mononuclear cells of SLE patients [19–21]. Also, an impaired EBV-specific T cell response is observed in SLE patients [19,30–32] and an increased serologic response has been demonstrated with high titers of antibodies to EBV antigens in SLE patients compared to healthy controls [29,33–45].

In this study, we substantiate previous results on serum FLC levels in SLE patients and show that elevated serum FLCs in SLE patients are associated with various serologic parameters and serve as a potential supplementary biomarker in SLE disease activity. Furthermore, we investigate if presence of serum FLCs associates with EBV antibodies in order to evaluate the role of EBV infection in the B cell hyperactivity in SLE patients.

## Materials and Methods

### Patients and controls

Serum samples were obtained from 45 unrelated Danish SLE patients attending the Department of Infectious Diseases and Rheumatology, Rigshospitalet, Copenhagen University Hospital, Denmark. All patients fulfilled the American College of Rheumatology classification criteria in SLE [8]. Serum samples from 40 non-medicated healthy controls, were obtained from personnel at Statens Serum Institut, Copenhagen, Denmark.

SLE patients suffering from cancer were not included in the cohort.

Six of the 51 SLE patients were found to have low eGFR (estimated glomerular filtration rate) (<60 ml/min/1.73m^2^), indicating renal insufficiency and were excluded from the study. eGFR was calculated from serum creatinine concentrations, age and gender of included patients, by the use of the following equation: eGFR (ml/min/1.73m^2^) = 175 x (creatinine (μmol/l)/88.4)^-1.154^ x (age)^-0.203^ x genderfactor (0.742 for women and 1 for men).

### Ethics statement

Approval from the Scientific-Ethical Committee of the Capital Region of Denmark was obtained (H-A-2007-0114). All participating subjects provided informed consent. All SLE patients provided written consent and healthy controls were used anonymously and therefore no written consent was necessary.

### Quantitative nephelometric assay

Serum Igs and FLC concentrations were quantified by nephelometry on a BN ProSpec (Siemens, Marburg, Germany). Total serum IgG, IgA and IgM concentrations were quantified by nephelometric test kits (Siemens Diagnostics, Marburg, Germany) and serum FLC concentrations were measured by the use of N Latex FLC kappa and N Latex FLC lambda kit (Siemens Diagnostics, Marburg, Germany) according to the manufacturer’s instructions. Normal ranges were defined as: 5,7–26,3 mg/l (λFLC), 3,3–19,4 mg/l (κFLC), 0,26–1,65 (FLC κ:λ ratio) [46].

### ELISA

EBV antibodies of all SLE patients and 20 of the 40 healthy controls, comprising antibodies to EBNA-1, EBV-VCA and EBV-EA/D, were previously measured by ELISA [33]. Furthermore, antibodies to EBNA-1, EBV-VCA and EBV-EA/D for the last 20 healthy controls were determined in this study. Antibodies against EBNA-1 and EBV-VCA were detected using ELISA test kits (Demeditec Diagnostics, Kiel-Wellsee, Germany) according to the manufacturer’s instructions as previously described [33]. Antibodies against EBV-EA/D were quantified with a new assay compared to the previous assay [33]. The new assay differed from the previously utilized by the use of NUNC polysorp microtitre plates (Thermo Fisher Scientific, Roskilde, Denmark) and in the serum samples used for the standard curve. However, comparison between the results should be valid as we only study antibody positive/negative samples and not antibody titers.

### Statistical analysis

Comparisons of concentrations of serum FLCs were performed using the unpaired nonparametric two-tailed Mann-Whitney test and considered significant at p-values less than 0.05. Data are presented with median values with statistical significant differences indicated with *, ** or *** for p-values less than 0.05, 0.01 or 0.001, respectively. Univariate correlation analyses were performed using Spearman correlation test for nonparametric data sets. All of the above mentioned data analyses were carried out using GraphPad Prism Software 5 (GraphPad Software Inc, La Jolla, CA, USA).

Furthermore, a comparison of EBV antibody seropositivity between SLE patients and healthy controls and also the possible association between elevated FLC concentrations and EBV antibody seropositivity were calculated using chi-squared test (with Yates’ correction) employing S-PLUS (Mathsoft, Inc, Seattle, WA, USA). These analyses were based on comparison of absolute numbers of SLE/healthy controls positive for EBV antibodies and with elevated FLC concentrations (λFLC and/or κFLC concentration above the normal range). Values below 0.05 were considered statistically significant and p-values below 0.00005 is presented as ~0.

## Results

### Characteristics of SLE patients and healthy controls

The average age for included SLE patients was 38.4 years ranging from 22 to 65 years as outlined in Table 1. For the healthy controls the average age was 38.5 years with a range of 25 to 72 years. The percentage of females was 96% in the SLE patient cohort and 78% in the healthy control group. The average SLEDAI score in SLE patients was 4.7 ranging from 0 to 21 and the average time since diagnosis was 10 years ranging from 0 to 24 years. The percentages positive for antinuclear autoantibodies (ANA) and dsDNA antibodies were 80 and 47 for included SLE patients, respectively, and 7.5 and 5 for the healthy controls. Table 1 shows more details on SLE patients and healthy controls.

**Table 1:** Characteristics of SLE patients and healthy controls.

	SLE patients	Healthy controls
No. of individuals	45	40
Average age (years) [range]	38.4 [22–65]	38.5 [25–72]
% females	96	78
% ANA-positive	80	7.5
% dsDNA antibody-positive	47	5
% Rheumatoid factor-positive		
IgM	16	ND
IgA	18	ND
Average amount of Ig’s (g/L) [range]:		
IgG	12.5 [5.8–24.1]	12.1 [8.3–18.7]
IgA	2.6 [<0.05–6.5]	2.7 [1.0–5.2]
IgM	1.1 [0.2–5.5]	1.7 [0.6–4.2]
% on immunosuppressive medication	67	0
Average C-reactive protein (mg/L) [range]	3.9 [0–21]	ND
% with low C3	51	ND
% with low C4	67	ND
Average serum creatinine (mg/dl)	0.61	ND
Average eGFR (ml/min/1.73m^2^)	117.38	ND
Average disease duration (years) [range]	10 [0–24]	ND
Average SLEDAI score [range]	4.7 [0–21]	ND

SLE—systemic lupus erythematosus, SLEDAI—SLE disease activity index, ANA- nuclear antibodies, dsDNA—double stranded DNA, Ig—immunoglobulin, eGFR—estimated glomerular filtration rate, ND—not determined.

### Prevalence of serum FLCs in SLE patients and healthy controls

**Fig 1:**
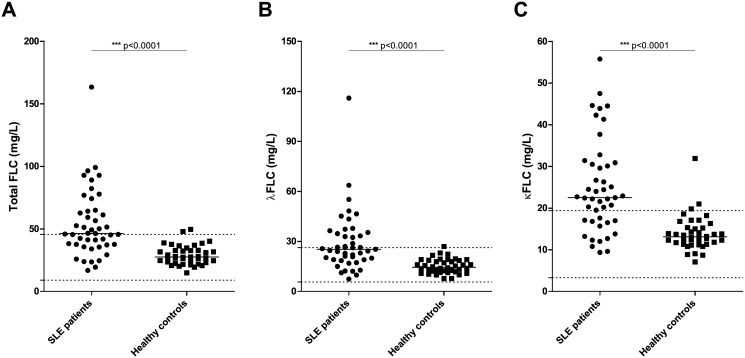
Concentration of serum FLCs in SLE patients and healthy controls. Total FLC (A), λFLC (B) and κFLC (C) levels in SLE patients (n = 45) and healthy controls (n = 40) measured by quantitative nephelometry. SLE patients suffering from renal insufficiency (eGFR<60 ml/min/1.73m^2^) were excluded. Middle horizontal bars represent median and statistical significant differences are indicated with *, ** or *** for p-values less than 0.05, 0.01 or 0.001. p-values for comparison of FLC levels in SLE patients and healthy controls are <0.0001. Maximum and minimum values of the normal ranges of λFLCs and κFLCs are indicated on the y-axis as dotted lines. FLCs—free light chains, SLE—systemic lupus erythematosus.

Sera from 45 SLE patients and 40 healthy controls were examined by quantitative nephelometry for concentration of κFLCs and λFLCs. As illustrated in Fig 1A, a significantly elevated concentration of total serum FLCs was found in SLE patients compared to healthy controls (p<0.0001). The median total FLC concentration was 46.4 mg/L in SLE patients compared to a median level of 27.6 mg/L in healthy controls. Similar results were obtained when examining the λFLC and κFLC levels individually (Fig 1B and 1C), with significantly higher concentrations in SLE patients compared to healthy controls (p<0.0001 in both cases). The median value of λFLCs was 25.3 mg/L in SLE patients and 14.5 mg/L in healthy controls, and the median value was 22.5 mg/L and 13.1 mg/L of κFLCs in SLE patients and healthy controls, respectively.

Sixty nine percent (31 of 45) of examined SLE patients had an elevated level of serum κFLCs above the normal range compared to 7.5% (3 of 40) of examined healthy controls. Similarly, 44% (20 of 45) of SLE patients compared to 2.5% (1 of 40) of healthy controls showed λFLC levels above the normal range.

Four of the 40 included healthy controls were found positive for ANA and/or anti-dsDNA, but none of these healthy controls were found to have elevated concentrations of serum FLCs.

A strong statistically significant difference in FLC concentrations was still observed between SLE patients and healthy controls after adjusting for total IgG, total IgA or total IgM concentrations, respectively (p<0.0001, regarding total FLC and λFLC and κFLC, individually, when adjusting for either total IgG, total IgA or total IgM, respectively), which was also expected as serum Ig levels were found to be similar between the two groups (Table 1).

To ensure that the elevated amount of serum FLCs in SLE patients was not due to the presence of a monoclonal (M) component, all serum samples were examined by serum protein electrophoresis and no M components was found in sera from SLE patients or from the healthy controls (results not shown). Furthermore, the ratio of κ:λ was, for the majority of individuals (All SLE patients and 38 of 40 healthy controls), within the normal range (results not shown), as expected in the absence of an M component.

### Correlation between serum FLC concentrations and global and clinical disease activity scores of SLE patients

**Fig 2:**
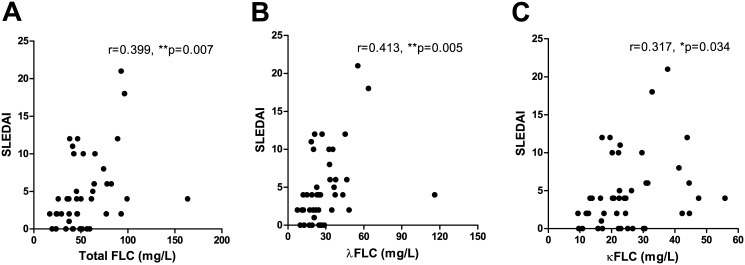
Correlation between serum FLC concentrations and SLEDAI scores of SLE patients. Correlation between SLEDAI scores and total FLC (A), λFLC (B) and κFLC (C) levels in SLE patients (n = 45). r-values are 0.399 (p = 0.007), 0.413 (p = 0.005) and 0.317 (p = 0.034) in A, B and C, respectively. FLCs—free light chains, SLE—systemic lupus erythematosus, SLEDAI—SLE disease activity index.

Total serum FLC concentrations correlated with global disease activity scores determined by the SLEDAI scores of the SLE patients (Fig 2A) with a calculated Spearman’s correlation coefficient (r) of 0.399 (p = 0.007). As illustrated in Fig 2B and 2C, similar results were obtained for individual levels of λFLC and κFLC (r-values at 0.413 (p = 0.005) and 0.317 (p = 0.034), respectively).

Similar results were obtained after adjusting for renal function in several ways (determined by serum creatinine concentrations or eGFR, respectively) with statistically significant correlations, (except between eGFR-adjusted κFLC and SLEDAI scores) (r-values of 0.566 (p<0.0001), 0.564 (p<0.0001) and 0.476 (p = 0.0005) when correlating SLEDAI scores with creatinine-adjusted concentrations of total FLC, λFLC and κFLC, respectively, and r-values of 0.320 (p = 0.022), 0.367 (p = 0.008) and 0.274 (p = 0.052) when correlating SLEDAI scores with eGFR-adjusted concentrations of total FLC, λFLC and κFLC, respectively) (results not shown).

Clinical SLEDAI scores (cSLEDAI, i.e. SLEDAI score excluding anti-dsDNA, complement, thrombocyte and leukocyte levels) were found to correlate with total FLC concentrations and λFLC concentrations of SLE patients (r = 0.358 (p = 0.016) and r = 0.330 (p = 0.027)), but not with κFLC (r = 0.232, p = 0.126). However, only a tendency of higher cSLEDAI scores were observed in SLE patients with elevated FLC levels (n = 31) compared to SLE patients with normal FLC levels (n = 14) (p = 0.111) (results not shown).

In comparison with the latter, SLE patients positive for anti-dsDNA did not have elevated cSLEDAI scores compared to SLE patients negative for anti-dsDNA (p = 0.234). Also, no correlation was observed between anti-dsDNA titres and cSLEDAI scores (r = 0.247, p = 0.102) (however, 12 SLE patients had a titer of >200 U/ml making the correlation test inconclusive). Similarly, SLE patients with complement consumption (low C3 or low C4) did not have higher cSLEDAI scores than SLE patients with normal complement levels (p = 0.201 and p = 0.688, regarding C3 and C4, respectively), and cSLEDAI and C-reactive protein (CRP) did not correlate (r = -0.050, p = 0.742) (results not shown).

### Serum FLC concentrations in relation to autoantibodies in SLE patients

**Fig 3:**
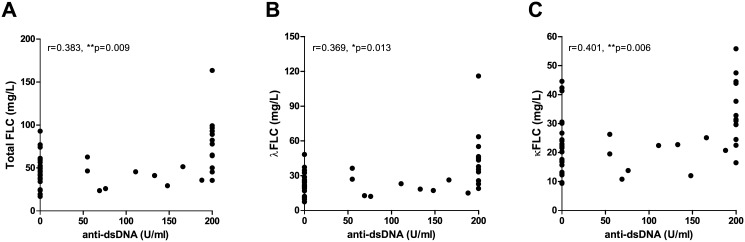
Correlation between serum FLC concentrations and anti-dsDNA titer. Correlation between anti-dsDNA titer (U/ml) and total FLC (A), λFLC (B) and κFLC (C) levels in SLE patients (n = 45). r-values are 0.383 (p = 0.009), 0.369 (p = 0.013) and 0.401 (p = 0.006) in A, B and C, respectively. FLCs—free light chains, SLE—systemic lupus erythematosus, dsDNA—double stranded DNA.

**Fig 4:**
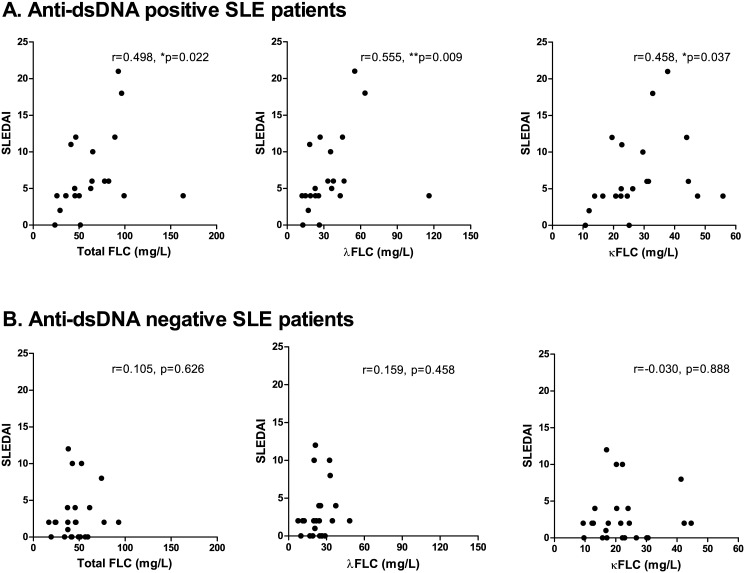
Correlation between serum FLC concentrations and SLEDAI scores in anti-dsDNA positive SLE patients. Correlation between SLEDAI scores and total FLC, λFLC and κFLC levels in anti-dsDNA positive SLE patients (A) (n = 21) and in anti-dsDNA negative SLE patients (B) (n = 24). **A: Anti-dsDNA positive SLE patients**. r-values are 0.498 (p = 0.022), 0.555 (p = 0.009) and 0.458 (p = 0.037) regarding total FLC, λFLC and κFLC levels, respectively. **B: Anti-dsDNA negative SLE patients**. r-values are 0.105 (p = 0.626), 0.159 (p = 0.458) and 0.030 (p = 0.888) regarding total FLC, λFLC and κFLC levels, respectively. FLCs—free light chains, SLE—systemic lupus erythematosus, SLEDAI—SLE disease activity index.

Concentrations of total FLC, λFLC and κFLC correlated with titer values of anti-dsDNA in the SLE patients (r values of 0.383 (p = 0.009), 0.369 (p = 0.013) and 0.401 (p = 0.006), respectively) (Fig 3), even though 12 of 45 SLE patients had a titer of >200 U/ml making the test inexact. However, no difference in FLC concentrations were observed when stratifying SLE patients by seropositivity of dsDNA antibodies. A statistically significant positive correlation was obtained between FLC levels and SLEDAI scores for dsDNA antibody-positive SLE patients (n = 21) (SLEDAI range: 0–21, median: 5) (Fig 4A) (r-values of 0.498 (p = 0.022), 0.555 (p = 0.009) and 0.458 (p = 0.037), regarding concentrations of total FLC, λFLC and κFLC, respectively). Conversely, this correlation was not achieved between FLC concentrations and SLEDAI scores for dsDNA antibody-negative SLE patients (n = 24) (SLEDAI range: 0–12, median: 2) (Fig 4B) (r-values of 0.105 (p = 0.626), 0.159 (p = 0.458) and -0.030 (p = 0.888), regarding concentrations of total FLC, λFLC and κFLC, respectively).

No associations were observed between rheumatoid factor (RF) IgA or RF IgM and serum FLC concentrations when stratifying SLE patients according to RF seropositivity (results not shown). However, the group of seropositive SLE patients with RF IgA and/or RF IgM was very small (n = 8 and n = 7, respectively) (Table 1) making conclusions unreliable.

### Serum FLC concentrations in relation to immunological and hematological markers

**Fig 5:**
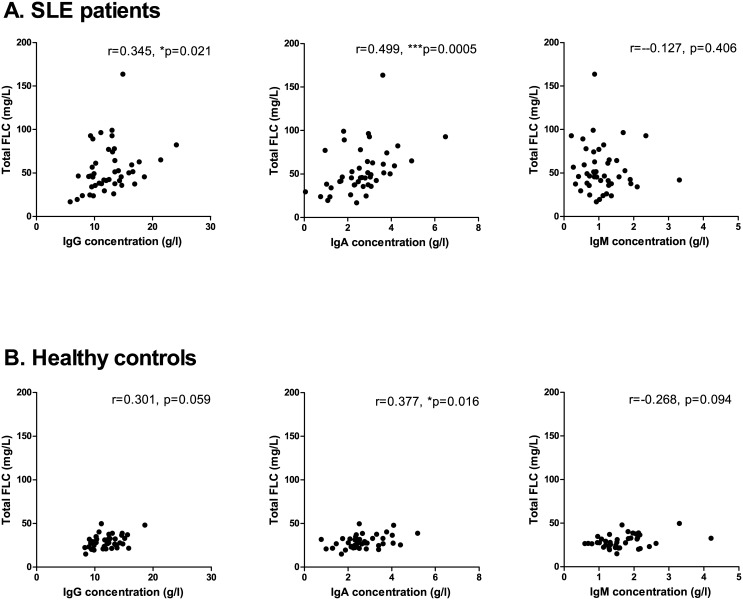
Correlation between serum FLC concentrations and total immunoglobulin levels. Correlation between total immunoglobulin levels and total FLC, λFLC and κFLC levels in SLE patients (A) (n = 45) and healthy controls (B) (n = 40) **A: SLE patients**. r-values are 0.345 (p = 0.021), 0.499 (p = 0.0005) and -0.127 (p = 0.406) regarding total FLC, λFLC and κFLC levels, respectively. **B: Healthy controls**. r-values are 0.301 (p = 0.059), 0.377 (p = 0.016) and -0.268 (p = 0.094) regarding total FLC, λFLC and κFLC levels, respectively. FLCs—free light chains, SLE—systemic lupus erythematosus.

A positive correlation was observed between serum FLC levels and total IgG concentration, total IgA concentration, but not total IgM concentrations in SLE patients (Fig 5A) (r-values of 0.345 (p = 0.021), 0.499 (p = 0.0005) and -0.127 (p = 0.406), respectively). Similar results were obtained for the healthy controls (Fig 5B) with a positive correlation between serum FLC levels and total IgA concentrations and a tendency of a positive correlation to total IgG concentrations (r-values of 0.301 (p = 0.059), 0.377 (p = 0.016) and -0.268 (p = 0.094), regarding total IgG, IgA and IgM, respectively).

**Fig 6:**
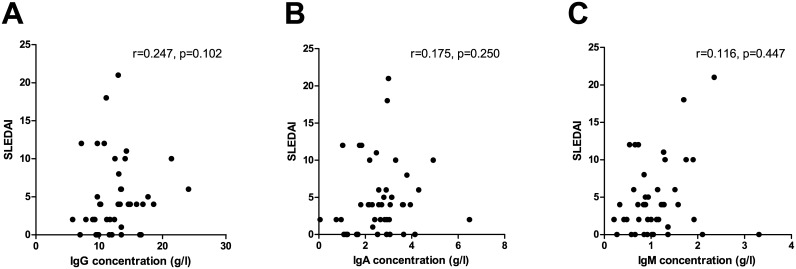
No correlation between SLEDAI scores and total immunoglobulin concentrations in SLE patients. No correlation between SLEDAI scores and total IgG, IgA and IgM in SLE patients (n = 45) with r-values of 0.247 (p = 0.102), 0.175 (p = 0.250) and 0.116 (p = 0.316) regarding total IgG, IgA and IgM, respectively. SLEDAI—systemic lupus erythematosus disease activity index.

As illustrated in Fig 6, no correlation was found between SLEDAI scores of the SLE patients and total IgG, total IgA or total IgM concentrations, respectively (r-values of 0.247 (p = 0.102), 0.175 (p = 0.250) and 0.116 (p = 0.447), respectively). Similar results were observed when stratifying SLE patients according to their seropositivity of dsDNA antibodies (results not shown).

**Fig 7:**
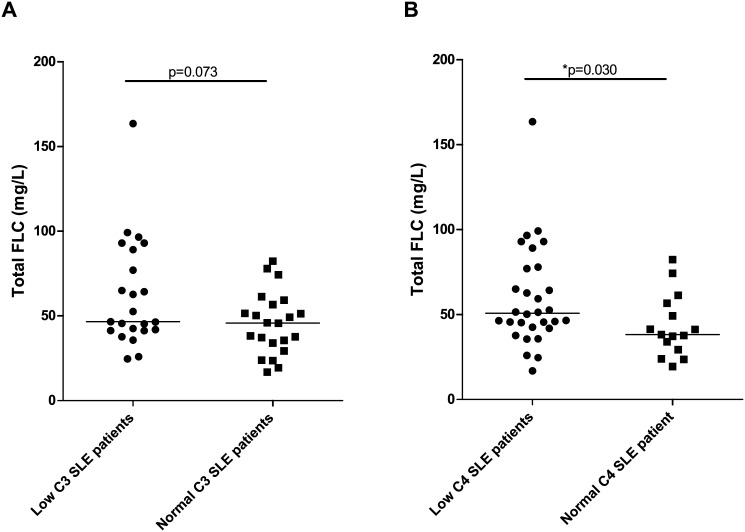
Concentrations of serum FLCs in SLE patients with low and normal levels of complement factors. Total FLC levels in SLE patients with low (n = 23)/normal (n = 22) C3 levels (A) (p = 0.073) and in SLE patients with low (n = 30)/normal (n = 15) C4 levels (B) (p = 0.030). Middle horizontal bars represent median and statistical significant differences are indicated with *, ** or *** for p-values less than 0.05, 0.01 or 0.001. FLCs—free light chains, SLE—systemic lupus erythematosus.

When comparing FLC concentrations of SLE patients with low levels (n = 23, and n = 30, respectively) and SLE patients with normal levels (n = 22, and n = 15, respectively) of the complement factors C3 and C4, respectively, an association was demonstrated between C4 complement consumption and elevated concentrations of serum FLCs (Fig 7) (p = 0.073 and p = 0.030 regarding C3 and C4 concentrations, respectively).

**Fig 8:**
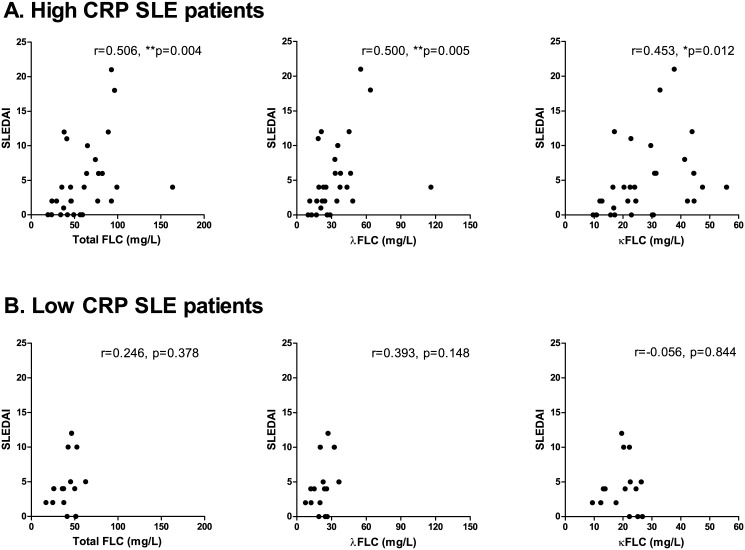
Correlation between serum FLC concentrations and SLEDAI scores in SLE patients with high CRP. Correlation between SLEDAI scores and total FLC, λFLC and κFLC levels in high CRP SLE patients (A) (n = 30) and in low CRP SLE patients (B) (n = 15). **A: High CRP SLE patients**. r-values are 0.506 (p = 0.004), 0.500 (p = 0.005) and 0.453 (p = 0.012) regarding total FLC, λFLC and κFLC levels, respectively. **B: Low CRP SLE patients**. r-values are 0.246 (p = 0.378), 0.393 (p = 0.148) and -0.056 (p = 0.844) regarding total FLC, λFLC and κFLC levels, respectively. FLCs—free light chains, SLE—systemic lupus erythematosus, SLEDAI—SLE disease activity index, CRP—C-reactive protein.

SLE patients stratified according to their concentrations of CRP showed that SLE patients with high CRP (>1 mg/l)(n = 30) had significantly higher serum λFLC concentrations compared to the SLE patients with low CRP (<1 mg/l) (n = 15) (p = 0.057, p = 0.050 and p = 0.059, regarding concentrations of total FLC, λFLC and κFLC, respectively) (results not shown). A positive correlation was obtained between FLC concentrations and SLEDAI scores for high CRP SLE patients (SLEDAI range: 0–21, median: 4) (Fig 8A) (r-values of 0.506 (p = 0.004), 0.500 (p = 0.005) and 0.453 (p = 0.012), regarding concentrations of total FLC, λFLC and κFLC, respectively), but not between FLC concentrations and SLEDAI scores for low CRP SLE patients (SLEDAI range: 0–12, median: 4) (Fig 8B) (r-values of 0.246 (p = 0.378), 0.393 (p = 0.148) and -0.056 (p = 0.844), regarding concentrations of total FLC, λFLC and κFLC, respectively).

No correlations were found between FLC concentrations and neither lymphocyte, leukocyte, nor thrombocyte levels (results not shown).

### Serum FLC concentrations and EBV antibodies

EBV antibodies of 45 SLE patients and 40 healthy controls, comprising antibodies against EBNA-1, EBV-VCA and EBV-EA/D were determined by ELISA. Percentages of SLE patients and healthy controls seropositive for these antibodies are listed in Table 2. When comparing absolute numbers of SLE patients and healthy controls seropositive for the various EBV antibodies results showed statistically significant differences between SLE patients and healthy controls regarding EBV-VCA IgA (p = 0.003), EBV-VCA IgM (p = 0.0007), EBV-EA/D IgG (p = 0.0001), EBV-EA/D IgA (p = ~0) and EBV-EA/D IgM (p = 0.0001) (Table 2).

**Table 2:** EBV antibodies in SLE patients and healthy controls (%).

	EBNA-1			EBV-VCA			EBV-EA/D		
	IgG	IgA	IgM	IgG	IgA	IgM	IgG	IgA	IgM
SLE patients (n = 45)	82	0	0	100	18	16	62	58	67
Healthy controls (n = 40)	75	0	2.5	82.5	5	5	18	13	23
p-values for comparison	NS	NS	NS	NS	0.003	0.0007	0.0001	~0	0.0001

EBV—Epstein-Barr virus, SLE—systemic lupus erythematosus, EBNA-1 –EBV nuclear antigen 1, EBV-VCA—EBV viral capsid antigen, EBV-EA/D—EBV early antigen diffuse, NS—not significant.

The relationship between elevated concentrations of serum FLC and EBV antibodies was examined demonstrating no significant associations.

Furthermore, no association was found between SLE patients/healthy controls and previous EBV infection (measured by VCA IgG), which was also expected as the majority of individuals (both SLE patients and healthy controls) included were found to have positive EBV serology.

## Discussion

In this study, the concentration of serum FLCs in SLE patients was quantified by nephelometry and the association with clinical parameters and EBV antibodies was investigated. The results showed significantly elevated levels of serum FLC in SLE patients that were associated with global disease activity scores, inflammatory status and B cell activity.

Elevated concentrations of FLC have previously been observed in both serum and urine from SLE patients [12–17] and in a number of other autoimmune diseases [14,47–49]. In this study, we confirm these previous reports showing statistically significant elevated concentrations of serum FLCs in SLE patients.

We observed that both κFLC and λFLC concentrations were elevated in sera from SLE patients compared to healthy controls with 69% compared to 7.5% having an elevated amount of κFLCs, and 44% compared to 2.5% having an elevated level of λFLCs. SLE patients with indications of renal insufficiency (eGFR<60 ml/min/1.73m^2^) were excluded from the study and the elevated concentrations of serum FLCs demonstrated were therefore not a result of decreased clearance. The κ:λ ratios were in this study found to be normal in the majority of individuals, indicating a polyclonal rather than a monoclonal B cell activation, also confirmed by the lack of detectable M components.

It would be expected that a very limited number of individuals in the healthy control group would have concentrations of FLCs above the normal range. 7.5% and 2.5% of healthy controls were found to have elevated levels of κFLC and λFLC, respectively. A larger percentage of the healthy controls included are men compared to the SLE patient cohort. However, when comparing FLC concentrations between the men and the women in the healthy control group, no significant difference was observed (results not shown). Hence, the difference in FLC concentrations observed between SLE patients and healthy controls is not a result of gender differences in the cohorts.

The high level of serum FLCs in SLE patients may be interpreted as reflecting an increased Ig synthesis in the B cells of the SLE patients but the elevated serum FLC levels were accompanied by normal levels of Igs (Table 1). Furthermore, a strong significant difference between SLE patients and healthy controls were still observed after adjusting FLC levels for total Ig concentrations, indicating that the elevated FLC concentrations demonstrated in SLE patients does not reflect an altered Ig production. Also, FLCs are measured in much lower concentration (mg/l) and have a short half life in the circulation (2–6 hours) [11–13] compared to Igs which are quantified in g/l and have a half life in the circulation of 5–25 days according to isotype [11], making small changes in the Ig synthesis hard to measure. Thus, FLC concentrations are possibly a better measure of the current B cell activity and Ig production status than total Ig measurements. Furthermore, previous studies by Hopper *et al*. have shown that secretion of Igs and FLCs may be independent processes by demonstrating that B cells are able to increase their secretion of FLCs independently of their antibody production, which support the current results [50].

The elevated serum FLCs were observed to correlate with a global disease activity score (SLEDAI) (also when adjusting FLC concentrations for renal function), and also with dsDNA antibody titers and with total IgG and total IgA concentrations confirming a strong connection between SLE disease activity and plasma cell productivity. This was further corroborated by the fact that FLC levels did not correlate with SLEDAI scores in dsDNA antibody-negative SLE patients.

Yet, SLEDAI scores of the SLE patients did not correlate with total serum Ig concentrations. These results demonstrate that serum FLC concentrations are a better indication of disease activity of SLE patients than total Ig measurements, which suggest that FLC levels might have a prospect for being an additional serologic biomarker in the clinical management of SLE patients without renal dysfunction, as also suggested by Aggarwal *et al* [15] and Jolly *et al* [16]. The potential of serum FLCs will need further investigations in larger SLE cohorts and in other patient groups in order to examine the specificity in relation to other inflammatory diseases. Longitudinal studies would additionally reveal if FLC concentrations could possibly also function as a biomarker for predicting future SLE disease activity flares and if the serum FLC analysis is more sensitive than measurements of complement factors and anti-dsDNA.

Serum FLC concentrations associated with the inflammatory status of the SLE patients. SLE patients with high CRP had elevated concentrations of λFLC in serum and furthermore, a positive correlation was obtained between FLC concentrations and SLEDAI scores of these high CRP SLE patients, but not of low CRP SLE patients. In addition, serum FLC concentrations associated with low levels of C4. Thus, elevated serum FLCs and increased disease activity reflected inflammation and complement consumption.

No link was found between cSLEDAI scores of the SLE patients and neither serum FLC levels (yet λFLC concentrations correlated with cSLEDAI scores), anti-dsDNA, complement consumption or CRP, indicating that none of these serological markers relate to the clinical manifestations of SLE in this cohort. However, as serum FLCs associate with the both anti-dsDNA titer, low complement and CRP, a connection to the serologic indications for active disease of SLE patients is suggested.

SLE has been linked to EBV and numerous research groups have demonstrated that SLE patients have an abnormal high viral load, indicating difficulties with controlling the latent EBV infection [18–21]. It could be speculated that the observed B cell activity or hyperactivity might be associated with an active EBV infection as latently infected memory B cells undergo differentiation into antibody (IgG and IgA)-secreting plasma cells during reactivation [51]. In agreement with this, FLC concentrations correlated with total IgG and total IgA, but not with total IgM concentrations in SLE patients, and with total IgA and a tendency for total IgG but not for IgM in healthy controls. The demonstrated increased seropositivity of EBV-directed antibodies in SLE patients did not associate with the elevated FLC concentrations. An explanation for this could be that serum FLC concentrations reflect the current EBV activity (reactivation) whereas EBV-directed antibodies reflect the extent of previous infection/reactivations. Concurrent measurements of EBV viral load in PBMCs (peripheral blood mononuclear cells) and serum FLC concentrations in longitudinal samples from SLE patients could clarify this hypothesis.

In conclusion, serum FLC concentrations were elevated in SLE patients, especially κFLC levels with 69% of SLE patients having abnormally high concentrations. As the concentration of FLCs correlated with global disease activity scores, reflected inflammation (high CRP and complement consumption) and B cell activity (high dsDNA antibody titers and total IgG and total IgA concentrations) in the SLE patients, the results obtained in this study suggest that the easily quantified FLC concentrations in the circulation could be a supplementary serologic biomarker in SLE.
