# Correction: Elevated Concentrations of Serum Immunoglobulin Free Light Chains in Systemic Lupus Erythematosus Patients in Relation to Disease Activity, Inflammatory Status, B Cell Activity and Epstein-Barr Virus Antibodies

**DOI:** 10.1371/journal.pone.0148151

**Published:** 2016-01-25

**Authors:** Anette H. Draborg, Magnus C. Lydolph, Marie Westergaard, Severin Olesen Larsen, Christoffer T. Nielsen, Karen Duus, Søren Jacobsen, Gunnar Houen

There are errors in the last sentence of the Materials and Methods under the subheading “Quantitative nephelometric assay.” The correct sentence should read: Normal ranges were defined as; 8,3–27,0 mg/l (λFLC), 6,7–22,4 mg/l (κFLC), 0,31–1,56 (FLC κ:λ ratio).

There are errors in the second paragraph of the Results under the subheading “Prevalence of serum FLCs in SLE patients and healthy controls.” The correct paragraph should read: Fifty three percent (24 of 45) of examined SLE patients had an elevated level of serum κFLCs above the normal range compared to 2.5% (1 of 40) of examined healthy controls. Similarly, 38% (17 of 45) of SLE patients compared to 2.5% (1 of 40) of healthy controls showed λFLC levels above the normal range.

There are multiple errors in the Discussion. The first sentence of the third paragraph should read: We observed that both κFLC and λFLC concentrations were elevated in sera from SLE patients compared to healthy controls with 53% compared to 2.5% having an elevated amount of κFLCs, and 38% compared to 2.5% having an elevated level of λFLCs. The second sentence of the fourth paragraph should read: 2.5% of healthy controls were found to have elevated levels of κFLC and λFLC, respectively. The first sentence in the last paragraph should read: In conclusion, serum FLC concentrations were elevated in SLE patients, especially κFLC levels with 53% of SLE patients having abnormally high concentrations.

Reference 46, Katzmann JA, Clark RJ, Abraham RS, Bryant S, Lymp JF, Bradwell AR et al. (2002) Serum reference intervals and diagnostic ranges for free kappa and free lambda immunoglobulin light chains: relative sensitivity for detection of monoclonal light chains. Clin Chem 48: 1437–1444. pmid:12194920 doi: 10.3410/f.717971156.793469463 should not appear in the published article. All subsequent references should be reduced by 1.

Fig 1 is incorrect in the published article. Please see the corrected version here.

**Fig 1:**
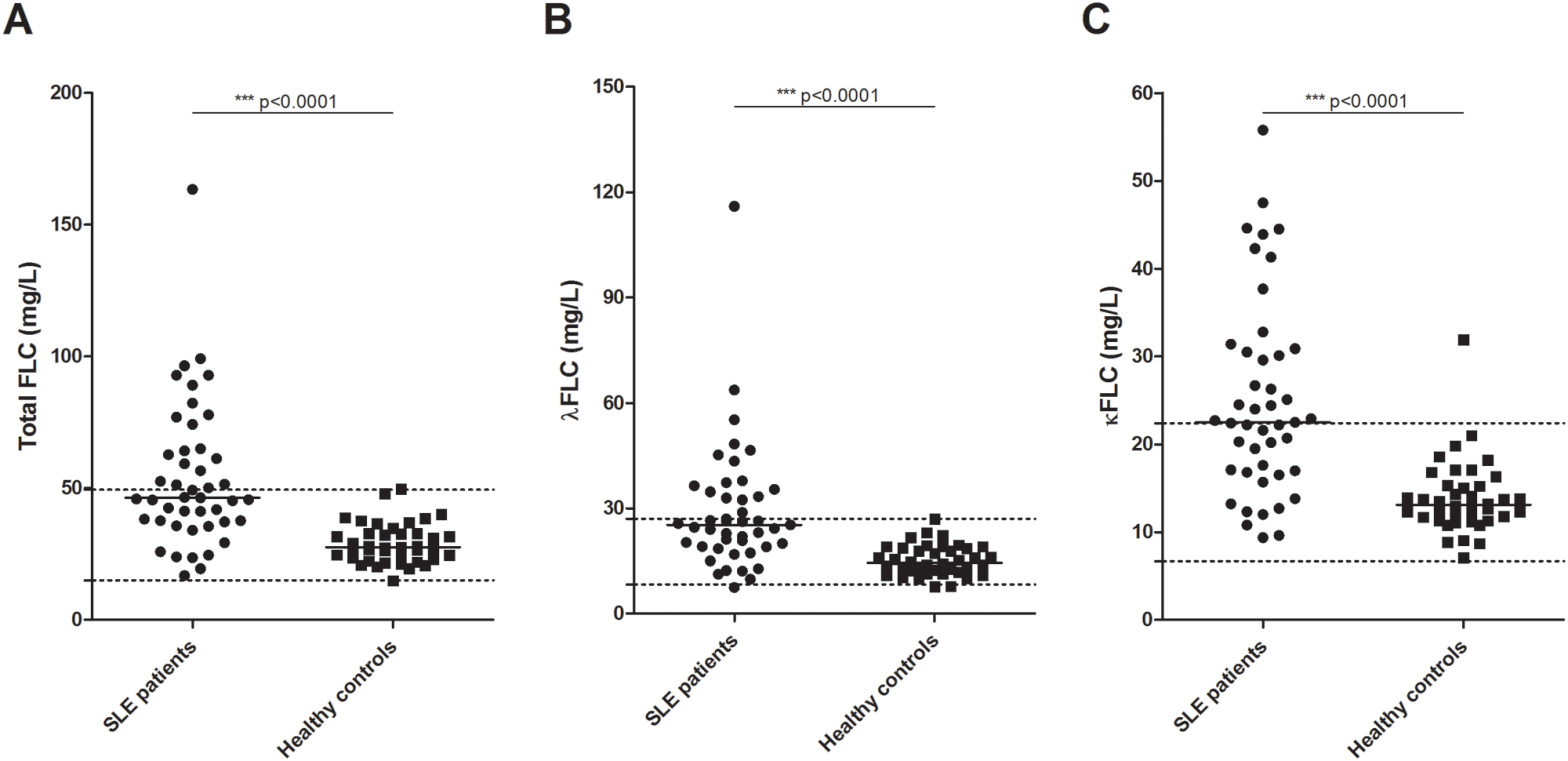
Concentration of serum FLCs in SLE patients and healthy controls. Total FLC (A), λFLC (B) and κFLC (C) levels in SLE patients (n = 45) and healthy controls (n = 40) measured by quantitative nephelometry. SLE patients suffering from renal insufficiency (eGFR<60 ml/min/1.73m2) were excluded. Middle horizontal bars represent median and statistical significant differences are indicated with *, ** or *** for p-values less than 0.05, 0.01 or 0.001. p-values for comparison of FLC levels in SLE patients and healthy controls are <0.0001. Maximum and minimum values of the normal ranges of λFLCs and κFLCs are indicated on the y-axis as dotted lines. FLCs—free light chains, SLE—systemic lupus erythematosus.
